# Priori Knowledge Makes Low-Light Image Enhancement More Reasonable

**DOI:** 10.3390/s25175521

**Published:** 2025-09-04

**Authors:** Zefei Chen, Yongjie Lin, Jianmin Xu, Kai Lu, Zihao Huang

**Affiliations:** School of Civil Engineering & Transportation, South China University of Technology, Guangzhou 510641, China; zefeichen@126.com (Z.C.); aujmxu@scut.edu.cn (J.X.); kailu@scut.edu.cn (K.L.); 202010101551@mail.scut.edu.cn (Z.H.)

**Keywords:** priori knowledge, priori channels, priori enhancement/suppression probability, GA Block

## Abstract

This paper presents a priori knowledge-based low-light image enhancement framework, termed Priori DCE (Priori Deep Curve Estimation). The priori knowledge consists of two key aspects: (1) enhancing a low-light image is an ill-posed task, as the brightness of the enhanced image corresponding to a low-light image is uncertain. To resolve this issue, we incorporate priori channels into the model to guide the brightness of the enhanced image; (2) during the enhancement of a low-light image, the brightness of pixels may increase or decrease. This paper explores the probability of a pixel’s brightness increasing/decreasing as its prior enhancement/suppression probability. Intuitively, pixels with higher brightness should have a higher priori suppression probability, while pixels with lower brightness should have a higher priori enhancement probability. Inspired by this, we propose an enhancement function that adaptively adjusts the priori enhancement probability based on variations in pixel brightness. In addition, we propose the Global-Attention Block (GA Block). The GA Block ensures that, during the low-light image enhancement process, each pixel in the enhanced image is computed based on all the pixels in the low-light image. This approach facilitates interactions between all pixels in the enhanced image, thereby achieving visual balance. The experimental results on the LOLv2-Synthetic dataset demonstrate that Priori DCE has a significant advantage. Specifically, compared to the SOTA Retinexformer, the Priori DCE improves the PSNR index and SSIM index from 25.67 and 92.82 to 29.49 and 93.6, respectively, while the NIQE index decreases from 3.94 to 3.91.

## 1. Introduction

Due to irreversible environmental factors and technical constraints, some photographs are often captured under suboptimal conditions, such as underexposure or overexposure [1]. This not only challenges human visual perception but also poses significant difficulties for more advanced image processing tasks, such as object detection [2], multi-object tracking [3], and instance segmentation [4]. Therefore, low-light image enhancement has been a prominent research focus. Low-light image enhancement is primarily achieved through two methods: (1) adjusting the camera parameters based on the environmental conditions before capturing, such as increasing the ISO, decreasing the shutter speed, and widening the aperture; (2) applying algorithms to map low-light images to reference ones after capturing, such as histogram equalization [5], gamma correction, and retinex [6].

Although existing methods enhance image brightness, they also introduce noise, blur, and artifacts in the enhanced images [7]. Plain methods, such as histogram equalization and gamma correction, often produce unnatural artifacts. This occurs because these methods naively apply enhancement functions to map low-light images to reference images, without considering the image as a whole. Land et al. [6] proposed the retinex hypothesis (a synthesis of the retina and cortex). According to this hypothesis, the observed image can be decomposed into two components: an illumination map, which represents the light intensity from the external environment, and a reflectance map, which corresponds to the intrinsic properties of the objects. Many researchers have applied the retinex theory to model the image decomposition process and recover the reflectance map [8,9]. However, due to limitations such as the capturing device and environmental conditions, the observed image often contains noise and distortions, which are subsequently propagated to the reflectance map during decomposition.

In the past decade, with the rapid development of computational power in computers, methods based on convolutional neural networks (CNNs) [10] have emerged as mushrooms after the rain [11,12]. Thanks to the powerful ability of convolutional neural networks, these methods demonstrated remarkable performance across a wide range of tasks. However, deep learning methods are data-driven, which limits their application in low-light image enhancement. With the emergence of paired image (low-light image and reference image) datasets, such as LOLv1 [13], LOLv2 [14], LSRW [15] and SICE [16], deep learning has started to be widely applied to low-light image enhancement tasks. Many researchers have attempted to use CNNs to approximate the decomposition process of the retinex theory [7,13,17,18,19]. In addition, GANs [20] have also been widely used in low-light image enhancement, such as pix2pix [21], CycleGAN [22], PD-GAN [23], and EnlightenGAN [24]. However, deep learning-based methods still suffer from noise, blur, and artifacts. To address this issue, we explain the reasons from the following three perspectives and propose corresponding improvements.

Firstly, in the process of low-light image enhancement, we intuitively assume that a pixel with higher brightness is more likely to be suppressed (become darker), while a pixel with lower brightness is more likely to be enhanced (become brighter), so as to achieve visual balance in the enhanced image. Once the structure and hyperparameters of the low-light image enhancement model are fixed, the enhancement/suppression probability of pixels is also fixed. Therefore, we refer to this probability as the pixel’s priori enhancement/suppression probability. Most existing methods are end-to-end black-box models [7,25], and their operational mechanisms are uninterpretable. Unlike end-to-end models, ref. Guo [1,26] propose using parameterized enhancement functions to individually map pixels in a low-light image, with parameters generated by a deep learning model. In [1,26], the priori enhancement probability of any pixel is fixed at 0.5, which is evidently unreasonable. To resolve this issue, we introduce an enhancement function that adaptively adjusts the priori enhancement probability based on pixel brightness. This ensures that, during the low-light image enhancement process, pixels with lower brightness have a higher priori enhancement probability, while those with higher brightness have a lower priori enhancement probability.

Secondly, in existing low-light image enhancement methods, the receptive field [27] of each pixel in the enhanced image is limited. This limitation causes the pixels to be inconsistent with the overall image, leading to issues such as noise, blur, and artifacts. Ref. [25] introduces the Squeeze-and-Excitation [28] into [29], proposing SE-Res2Net and using it as the backbone network for low-light image enhancement tasks. This method enhances pixel interaction in the enhanced image by averaging each channel and then multiplying it by the corresponding channel, thereby increasing the interaction between pixels to some extent. Recently, Carion et al. [30] introduced the Transformer [31] into computer vision tasks, proposing DETR. However, DETR computes interactions between the features at each position and all positions in the feature map, resulting in a substantial computational burden. Zhu et al. [32] incorporated the deformable convolutions [33,34] into the Transformer, significantly reducing the computational complexity. Inspired by this, this paper proposes the GA Block (Global-Attention Block). The GA Block first computes four reference points for each position in the feature map. Then, the weights of these reference points are calculated based on cosine similarity. Finally, the weighted sum of these features at the four reference points is computed to obtain the output feature at the corresponding position. Since the GA Block computes each pixel in the enhanced image based on all pixels in the low-light image, it alleviates issues such as noise, blur, and artifacts in the enhanced image.

Thirdly, since the relationship between abnormal images and reference images in low-light image enhancement tasks is many-to-many, the brightness of the enhanced images should be controllable. The brightness of the enhanced images generated by [13,24,35] is uncertain. Ref. Guo [1,26] maps arbitrary low-light images to enhanced images with an average brightness of 0.5. Refs. [7,19] incorporate the ratio of the brightness between low-light and reference images into the illumination adjustment process. To address this issue, we propose a priori channels to indicate the brightness of the enhanced image, which is then cross-concat with the low-light image. During the inference stage, the brightness of the enhanced image can be adjusted by modifying the priori channels. The code and experiments have been open-sourced at https://github.com/zefeichen/PrioriDCE (accessed on 18 August 2025).

The contributions of this paper are as follows:

1. This paper presents a parametric enhancement function in which the priori enhancement probability is adaptively adjusted based on pixel’s brightness.
2. This paper proposes GA Block, which allows each pixel in the enhanced image to be computed from all the pixels in the low-light image, making the enhanced image appear more natural.
3. This paper proposes a priori channels for indicating the brightness of the enhanced image, which allows the enhanced image to be freely adjusted.
4. This paper presents comprehensive experiments, and the results demonstrate that the proposed Priori DCE significantly enhances the quality of the enhanced images.

## 2. Related Works

### 2.1. Plain Methods

Histogram equalization transforms the probability density function of a low-light image into a uniform distribution using an enhancement function. Pizer et al. [36] proposed Adaptive Histogram Equalization (AHE). AHE divides the image into several subimages, such as multiple 8×8 subimages, and then applies histogram equalization to each subimage. Gamma correction maps a low-light image to a reference image using a gamma function. However, due to its simplicity, gamma correction has noticeable drawbacks, such as loss of detail, inappropriate exposure, and unnatural boundary enhancement. To overcome these drawbacks, Bennett et al. [37] enhanced each pixel in a low-light image by adjusting its virtual exposure. Yuan et al. [38] used an *S*-shaped curve with two parameters to enhance low-light images, automatically adjusting these two parameters based on the image’s brightness.

### 2.2. Retinex

Land et al. [6] first proposed a low-light image enhancement method based on the imaging principles of the camera, named retinex. The principle of retinex is to decompose the observed image into a reflectance map and an illumination map. Based on this principle, they first introduced SSR (Single-Scale Retinex). SSR estimates the illumination map by applying a Gaussian filter to the observed image and then calculates the reflectance map by deriving it from the observed image and the illumination map. Expanding upon SSR, Rahman et al. [39] introduced MSR (Multi-Scale Retinex), which involves estimating the illumination map through a Gaussian filter at various scales to enhance image details and textures. Nonetheless, akin to SSR, MSR encounters challenges with pronounced color casts in images. To address this issue, Jobson et al. [40] improved MSR and proposed MSRCR (Multi-Scale Retinex with Color Restoration). MSRCR builds upon MSR by introducing a color restoration factor *C*, to adjust for the color distortion caused by contrast enhancement in local regions of the image. Wang et al. [41] proposed using a dual logarithmic transformation function to map pixels, which helps balance detail and natural in the enhanced image.

### 2.3. Deep Learning

In recent years, the increasing computational power has led to the widespread application of deep learning-based methods in low-light image enhancement tasks. Given that the decomposition process in retinex theory remains unknown and deep learning models exhibit powerful fitting capabilities, Wei et al. [13] utilized a deep learning model to fit the decomposition process in the retinex theory and proposed RetinexNet. Jiang et al. [24] proposed EnlightenGAN, which employs both a local and a global discriminator to evaluate the local and global features of an image, thereby preserving its details and textures in the enhanced image. Cai et al. [42] incorporated the transformer [31] into the retinex model, proposing Retinexformer. As the attention mechanism in transformers is pixel-based, it considerably increases the model’s computational cost. To address this, Retinexformer modifies the attention mechanism from a pixel-based to a channel-based mechanism. However, whether using retinex theory-based methods or end-to-end methods, the essence of these approaches is to use deep learning to fit the mapping relationship from the low-light image to the reference image. Unlike the aforementioned models, Guo et al. [1] employ a series of parametric functions as enhancement functions, with a deep learning model generating the parameters for these functions. These enhancement functions are then applied to map the low-light image to the reference image. Although [1] is not fundamentally different from previous deep learning-based methods, this approach makes the low-light image enhancement process interpretable. However, the parametric functions used in the methods of [1] assign a priori enhancement probability of 0.5 to all pixels, which is clearly unreasonable.

## 3. Methodology

**Figure 1:**
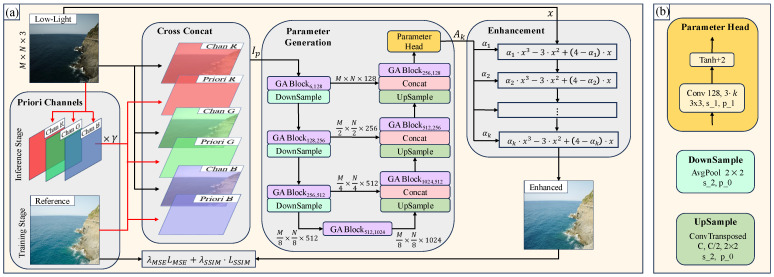
(**a**) The framework of Priori DCE. (**b**) Implementation details in (**a**).

In this section, we first present the framework and data flow of the Priori DCE, as illustrated in Figure 1a. Subsequently, we provide a detailed description of the model’s key components, including the parameter generation model (Section 3.2), the enhancement function (Section 3.3), and the training process (Section 3.4).

### 3.1. Pipeline

Since the photos taken of the same scene change with variations in lighting intensity, the relationship between an abnormal image and a reference image in the same scene is not one-to-one but rather many-to-many. Consequently, the input to Priori DCE comprises two components: the low-light image and the priori channels, as depicted on the left side of Figure 1a. The low-light image represents the scene information under abnormal lighting conditions, while the priori channels represents the desired brightness of the enhanced image. To achieve this goal, during the training stage, we use the channels information of the reference image as the priori channels. Unlike the training stage, the inference stage does not have a corresponding reference image, so we need to predefine the priori channels. Next, we first introduce the data flow during the training stage.

**Figure 2:**
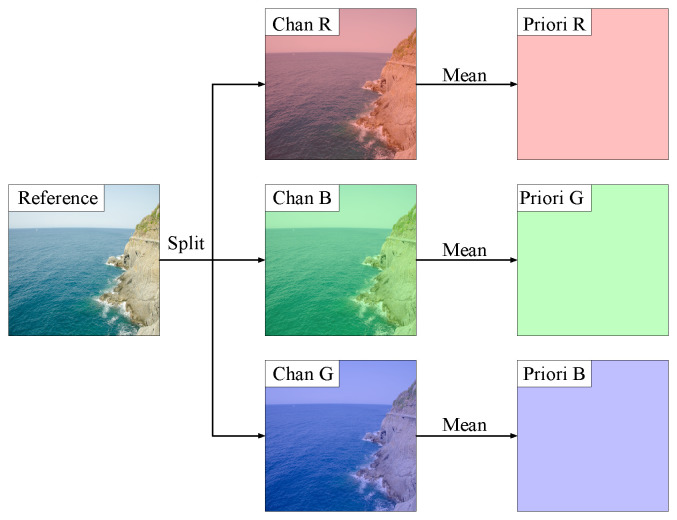
The process of deriving the priori channels during the training stage.

1. The reference image serves as the enhancement target for a low-light image of size M×N×3, and the process of deriving the priori channels from it is illustrated in Figure 2. The reference image is first segmented by channel into Chan R, Chan G, and Chan B, denoted as $\left[R,G,B\right],R,G,B\in R^{M\times N\times 1}$. Then, the mean value of each corresponding channel is calculated, and these priori channels (Priori R, Priori G, and Priori B) are generated based on these mean values, denoted as $\left[R_{p},G_{p},B_{p}\right],R_{p},G_{p},B_{p}\in R^{M\times N\times 1}$. This process can be represented by Equation (1).
  (1) $$C_{p}=mean\left(C\right),C\in \left[R,G,B\right].$$
2. Cross-concat takes the low-light image and the priori channels as inputs and merges them in the order shown in Figure 1a. The merged result is denoted as $I_{p},I_{p}\in R^{M\times N\times 6}$, and the merging process is illustrated in Equation (2).
  (2) $$I_{p}=Concat\left(\left[R,R_{p},G,G_{p},B,B_{p}\right]\right)$$
3. The parameter generation model processes $I_{p}$, generating parameters $A_{k}$ that correspond to a set of enhancement functions for each pixel in the low-light image.
4. The low-light image is enhanced using the obtained enhancement functions to generate the corresponding enhanced image.
5. Calculate the loss value between the enhanced image and the reference image, then perform backpropagation and update the parameters in the parameter generation model.

During the inference stage, in order to enhance the low-light image to the corresponding brightness, the low-light image is first split by channels, and then the average value of each channel is calculated to obtain the corresponding Chan R, Chan G, and Chan B. Finally, the Chan R, Chan G, and Chan B are scaled by a factor of γ to serve as prior channels, as shown in the inference stage of Figure 1a.

### 3.2. Parameter Generation

**Figure 3:**
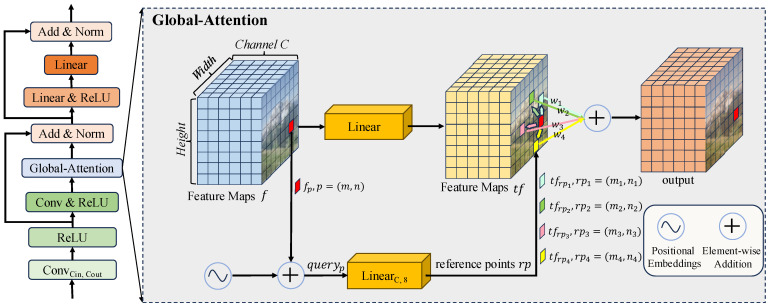
Global-Attention Block.

The parameter generation model is based on UNet [43] and comprises DownSample, UpSample, Concat, Parameter Head, and GA ${\mathrm{Block}}_{Cin,Cout}$. Figure 1b shows the details of DownSample, UpSample, and Parameter Head. DownSample is an average pooling layer with a kernel size of 2×2, stride of 2, and padding of 0. Its primary role is to perform 2× downsampling on the feature map, thereby increasing the model’s receptive field. UpSample is a deconvolution layer with input channels of *C*, output channels of $\frac{C}{2}$, a kernel size of 2×2, a stride of 2, and padding of 0. Its primary role is to double the height and width of the feature map while reducing the channels to half of the original. The role of Concat is to combine feature maps from multiple scales. The Parameter Head comprises a convolutional layer and an activation layer (Tanh+2). The convolutional layer has input channels of 128, output channels of 3×k, a kernel size of 3×3, a stride of 1, and padding of 1, where *k* denotes the number of iterations of the enhancement function. This layer adjusts the channels of the output feature map based on the number of iterations of the enhancement function. The activation layer then scales the output feature map to the range [1,3]. GA ${\mathrm{Block}}_{Cin,Cout}$ refers to a block where the input is a feature map with Cin channels and the output is a feature map with Cout channels. Figure 3 illustrates the structure of GA ${\mathrm{Block}}_{Cin,Cout}$ on the left and the core GA on the right. In Figure 3, Conv/${\mathrm{Linear}}_{Cin,Cout}$ indicates that the input to the Conv/Linear layer is a feature map with Cin channels, and the output is a feature map with Cout channels. A Conv/Linear layer without special notation implies that both the input and output feature maps have the same size and channels. Notably, GA ${\mathrm{Block}}_{Cin,Cout}$ alters only the channels in the feature map (from Cin to Cout), without modifying its size.

GA is the core component of GA ${\mathrm{Block}}_{Cin,Cout}$, as depicted on the right side of Figure 3. To illustrate the data processing procedure of GA, we use the feature $f_{p}$ at the red point *p* with coordinates (m,n) as an example. The processing steps are outlined as follows.

1. A linear transformation of $f_{p}$ is performed using the fully connected layer Linear (top) to obtain $tf_{p}$, as shown in Equation (3).
  (3) $$tf_{p}=Linear\left(f_{p}\right)$$
2. The position of the feature $f_{p}$ is encoded [32], and then the result of the position encoding is added to $f_{p}$ to obtain $query_{p}$, as shown in Equation (4).
  (4) $$query_{p}=PosEmb\left(f_{p}\right)+f_{p}$$
3. The fully connected layer ${\mathrm{Linear}}_{C,8}$ (below) is used to linearly transform $query_{p}$, mapping the $query_{p}$ with *C* channels to an positional feature rp with 8 channels, where $rp=[m_{1},n_{1},m_{2},n_{2},m_{3},n_{3},m_{4},n_{4}]$, as shown in Equation (5). rp corresponds to four reference points related to point *p*, namely the cyan point $rp_{1}\left(m_{1},n_{1}\right)$, the green point $rp_{2}\left(m_{2},n_{2}\right)$, the pink point $rp_{3}\left(m_{3},n_{3}\right)$, and the yellow point $rp_{4}\left(m_{4},n_{4}\right)$.
  (5) $$rp=Linear_{C,8}\left(query_{p}\right)$$
4. The weight $w_{i}$ of the feature $tf_{rp_{i}}$ is calculated based on the cosine similarity between the features $tf_{rp_{i}}$ and the feature $tf_{p}$, as shown in Equation (6).
  (6) $$w_{i}=\frac{e^{cos(tf_{p},tf_{rp_{i}})}}{\sum_{j}^{4}e^{cos(tf_{p},tf_{rp_{j}})}}$$
5. Calculate the weighted sum of the four reference points to obtain the output feature at the red point *p*, as shown in Equation (7).
  (7) $$output_{p}=\sum_{i=1}^{4}w_{i}\cdot tf_{rp_{i}}$$

### 3.3. Enhancement Function

#### 3.3.1. Priori Probability

**Figure 4:**
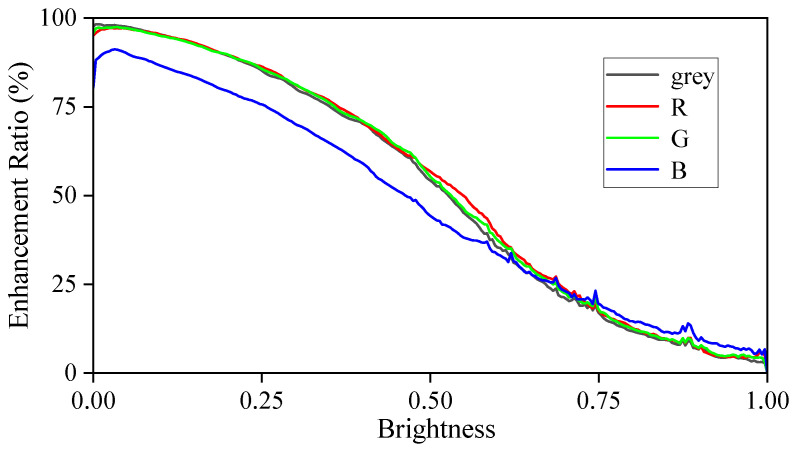
The ratio of pixels with different brightness that are enhanced during the process of transforming abnormal images to reference images in the SICE dataset.

Under conditions of excessively strong or weak lighting, photos may suffer from overexposure or underexposure. The SICE dataset [16] includes a variety of abnormal images taken under different lighting conditions, along with their corresponding reference images. Figure 4 demonstrates the pixel ratios in the grey, R, G, and B channels that require enhancement (brightening) as brightness changes during the transformation from abnormal images to reference images. It is evident that as pixels’ brightness increases, the proportion of pixels requiring enhancement decreases gradually. This suggests that during the enhancement process of abnormal images, a pixel with higher brightness should have a higher probability to be suppressed, while a pixel with lower brightness should have a higher probability to be enhanced.

The enhancement function is formed by a series of parameterized base functions that undergo multiple iterations. These base functions are denoted as $f_{\alpha }$, where α is a parameter and satisfies α∈I,I=[s,e]. For convenience, this paper denotes the enhancement function with *k* iterations as $f_{A_{k}}$, where $A_{k}=\left[\alpha_{1},\alpha_{2},\ldots ,\alpha_{i},\ldots ,\alpha_{k}\right]\in I^{k}$, and $\alpha_{i}$ is the parameter corresponding to the base function at the *i*-th iteration. The enhancement function maps the pixel’s brightness from *x* to *y*. The enhancement function obtained by iterating the basis function once or twice is shown in Equations (8) and (9), respectively, while the enhancement function with three or more iterations of the basis function follow a similar form.

(8) $$y=f_{A_{1}}\left(x\right)=f_{\alpha_{1}}\left(x\right)$$

(9) $$y=f_{A_{2}}\left(x\right)=f_{\alpha_{2}}\left(f_{\alpha_{1}}\left(x\right)\right)$$

$A_{k}$ is a *k*-dimensional space spanned over the interval *I*, and the measure $M_{A_{k}}$ represents the size of space $A_{k}$. For example, $A_{1}$ is a 1-dimensional space, and its corresponding measure $M_{A_{1}}$ is the length; $A_{2}$ is a 2-dimensional space, and its corresponding measure $M_{A_{2}}$ is the area; $A_{3}$ is a 3-dimensional space, and its corresponding measure $M_{A_{3}}$ is the volume. Each point in the space $A_{k}$ represents an enhancement function. For a pixel with brightness *x*, the space $A_{k}$ is divided into three subspaces based on whether the pixel is enhanced: (1) the subspace where $y=f_{A_{k}}\left(x\right)>x$, corresponding to an enhancement function that increases the pixel’s brightness; this subspace is denoted as $A_{k}^{+}$; (2) the subspace where $y=f_{A_{k}}\left(x\right)=x$, corresponding to an enhancement function that leaves the pixel unchanged; this subspace is denoted as $A_{k}^{0}$; (3) the subspace where $y=f_{A_{k}}\left(x\right)<x$, corresponding to an enhancement function that decreases the pixel’s brightness; this subspace is denoted as $A_{k}^{-}$. It is important to note that, whether increasing, leaving unchanged, or decreasing the pixel’s brightness, the overall goal is to improve the image’s quality.

Given that the parameter $\alpha_{i}$ is the output of the deep learning model, and the deep learning model is an inherently uninterpretable black-box model. Therefore, we assume that the probability of $\alpha_{i}$ taking any value in the interval *I* is equal, which means that the parameter $\alpha_{i}$ can be considered as a random variable following a uniform distribution on the interval *I*, denoted as $\alpha_{i}∼U\left(s,e\right)$. $A_{k}$ is the joint distribution of $\left(\alpha_{1},\alpha_{2},\ldots ,\alpha_{i},\ldots ,\alpha_{k}\right)$; thus, the probability density function of $A_{k}$ is given by Equation (10).

(10) $$p\left(A_{k}\right)=\frac{1}{M_{A_{k}}}$$

Based on the above analysis, the probability that a pixel with brightness *x* is enhanced, unchanged, or suppressed is shown in Equation (11).

(11) $$\begin{array}{c} p\left(A_{k}^{+}\right)=\frac{M_{A_{k}^{+}}}{M_{A_{k}}},\;p\left(A_{k}^{0}\right)=\frac{M_{A_{k}^{0}}}{M_{A_{k}}},\;p\left(A_{k}^{-}\right)=\frac{M_{A_{k}^{-}}}{M_{A_{k}}}\\ p\left(A_{k}^{+}\right)+p\left(A_{k}^{0}\right)+p\left(A_{k}^{-}\right)=1 \end{array}$$

$p(A_{k}^{+})$, $p(A_{k}^{0})$, and $p(A_{k}^{-}$) are solely dependent on the basis functions, which are determined and immutable. Consequently, these probabilities are termed the prior enhancement probability, priori unchanged probability, and priori suppression probability for a pixel with brightness *x*, respectively. For simplicity, we collectively refer to these probabilities as priori probabilities.

#### 3.3.2. The Shortcomings of the Current Method

Zero DCE employs a series of parameterized quadratic functions $g_{\alpha }$ as basis functions, as showed in Equation (12).

(12) $$y=g_{\alpha }\left(x\right)=\alpha \cdot x^{2}+\left(1-\alpha \right)\cdot x$$

where α∈I,I=[−1,1].

**Figure 5:**
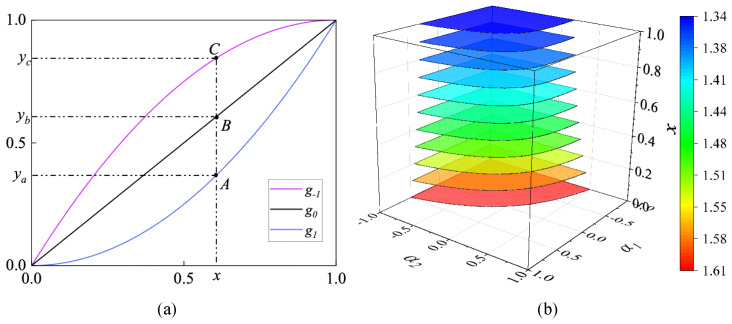
(**a**,**b**) illustrate the enhancement spaces of the enhancement functions $g_{A_{1}}$ and $g_{A_{2}}$, respectively.

Figure 5a illustrates the curves of three special basis functions $g_{-1},g_{0}$, and $g_{1}$, which also act as the enhancement functions when the basis functions are iterated once. The horizontal axis represents the original pixel’s brightness, while the vertical axis represents the enhanced brightness. Specifically, the enhancement function $g_{0}$ coincides with y=x. As shown in Figure 5a, for a pixel with an original brightness of *x*, when the parameter α gradually changes from −1 to 0, the enhanced brightness decreases from $y_{c}$ to $y_{b}\;\left(y_{b}=x\right)$; when the parameter α then gradually changes from 0 to 1, the enhanced brightness decreases further from $y_{b}$ to $y_{a}$. The space $A_{1}$ of parameter α is 1-dimensional, and its corresponding measure $M_{A_{1}}$ is a length of 2. At the same time, the measures of its corresponding subspaces $A_{1}^{+}$ and $A_{1}^{-}$ are both 1. Furthermore, the priori enhancement probability $p(A_{1}^{+})$ and priori suppression probability $p(A_{1}^{-})$ satisfy $p\left(A_{1}^{+}\right)\equiv p\left(A_{1}^{-}\right)\equiv 0.5$.

The enhancement function $g_{A_{2}}$ is derived by iterating the basis function $g_{\alpha }$ twice. At this point, the parameter space $A_{2}$ is a 2-dimensional plane, and the corresponding measure $M_{A_{2}}$ is a square with an area of 4. Figure 5b shows pixels with different brightness and the corresponding planar space $A_{2}$. The vertical axis (*x*-axis) represents the pixel’s brightness. The $A_{2}$ space is a parameter space composed of parameters $\alpha_{1}$ and $\alpha_{2}$. Surfaces of the same color are parallel to the $\alpha_{1}-\alpha_{2}$ plane, representing the subspace $A_{2}^{+}$ of a pixel with brightness *x*, while different colors represent the area of the corresponding surface. It can be seen that as we move upward along the vertical axis, the pixels’ brightness *x* gradually increases, and the area of the corresponding subspace $A_{2}^{+}$ decreases from 1.61 (red) to 1.34 (blue).

**Figure 6:**
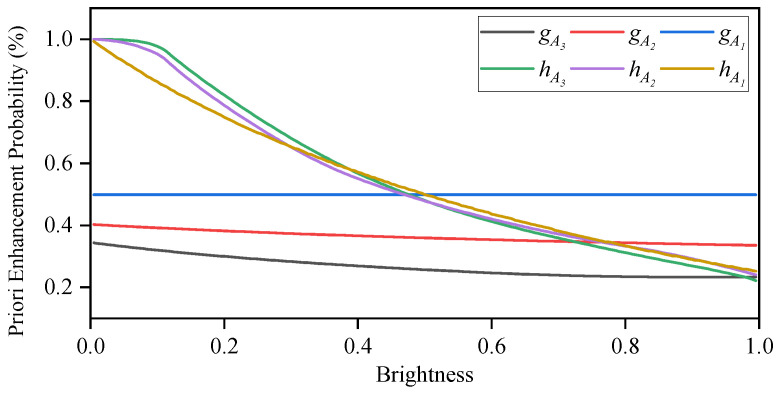
The prior enhancement probability of different enhancement functions.

Figure 6 illustrates the priori enhancement probabilitiy for the enhancement functions $g_{A_{1}}$, $g_{A_{2}}$, and $g_{A_{3}}$, respectively. It is observed that the priori enhancement probability of $g_{A_{1}}$ remains constant at 0.5, whereas the priori enhancement probabilities of $g_{A_{2}}$ and $g_{A_{3}}$ decrease as brightness increases. Overall, these priori enhancement probabilities remain below 0.5. In other words, the prior suppression probabilities for $g_{A_{2}}$ and $g_{A_{3}}$ consistently exceed their prior enhancement probabilities, which is unreasonable.

#### 3.3.3. Solutions

To solve the problem raised in Section 3.3.2, in this section, we propose using the cubic function $h_{\alpha }$ as the basis function, as shown in Equation (13).

(13) $$y=h_{\alpha }\left(x\right)=\alpha \cdot x^{3}-3\cdot x^{2}+\left(4-\alpha \right)\cdot x$$

where α∈I,I=[1,3].

**Figure 7:**
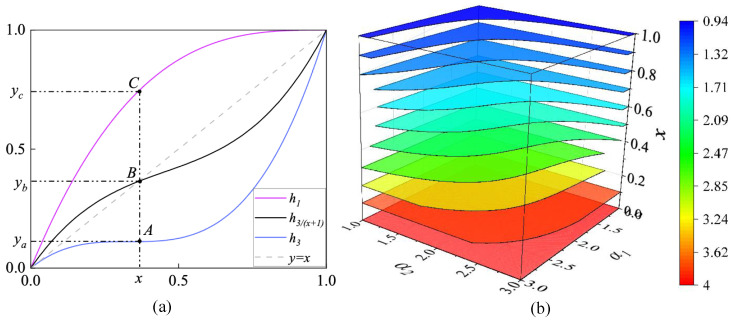
(**a**,**b**) illustrate the enhancement spaces of the enhancement functions $h_{A_{1}}$ and $h_{A_{2}}$, respectively.

Figure 7a shows the curves of these basis functions $h_{1}$, $h_{\frac{3}{x+1}}$, and $h_{3}$, respectively. It can be seen that for a pixel with brightness *x*, when the parameter α gradually increases from 1 to $\frac{3}{x+1}$, its enhanced brightness decreases from $y_{c}$ to $y_{b}$. During this process, the pixel’s brightness is enhanced. When the parameter α gradually increases from $\frac{3}{x+1}$ to 3, the enhanced brightness decreases from $y_{b}$ to $y_{a}$. During this process, the pixel’s brightness is suppressed. For a pixel with brightness *x*, its priori enhancement probability is $p\left(A_{1}^{+}\right)=\frac{1.5}{x+1}-0.5$. In other words, the priori enhancement probability of a pixel with brightness *x* is adaptive.

When the basis function $h_{\alpha }$ is iterated twice to obtain the enhancement function $h_{A_{2}}$, the corresponding parameter space $A_{2}$ forms a square with the area of 4. Similar to Figure 5b, Figure 7b shows the enhancement subspace $A_{2}^{+}$ corresponding to pixels with different brightness. It can be observed that as *x* increases, the area of the enhancement subspace $A_{2}^{+}$ gradually decreases from 4 (red) to 0.94 (blue).

Figure 6 shows these priori enhancement probabilities of the enhancement functions $h_{A_{1}}$, $h_{A_{2}}$, and $h_{A_{3}}$, respectively. It can be observed that as the pixel’s brightness gradually increases, these priori enhancement probabilities decrease from 1 to 0.

From the above analysis, it can be seen that ($g_{A_{1}}$, $g_{A_{2}}$, and $g_{A_{3}}$) is more suitable as an enhancement function than ($h_{A_{1}}$, $h_{A_{2}}$, and $h_{A_{3}}$).

### 3.4. Training

The Structural Similarity Index Measure (SSIM) quantifies the similarity between two different images in terms of brightness, contrast, and structure. When SSIM is used as a loss function, it is defined by Equation (14):

(14) $$L_{SSIM}=1-\sum_{j=1}^{J}\frac{\left(2\mu_{I_{j}}\mu_{I_{j}^{\prime }}+c_{1}\right)\left(\sigma_{I_{j},I_{j}^{\prime }}+c_{2}\right)}{\left(\mu_{I_{j}}^{2}+\mu_{I_{j}^{\prime }}^{2}+c_{1}\right)\left(\sigma_{I_{j}}^{2}+\sigma_{I_{j}^{\prime }}^{2}+c_{2}\right)}$$

where *j* represents the index of an image, while *J* denotes the total number of images. $I_{j}$ refers to the low-light image, and $I_{j}^{\prime }$ corresponds to the enhanced image. $\mu_{I_{j}}$ and $\mu_{I_{j}^{\prime }}$ are the mean values of $I_{j}$ and $I_{j}^{\prime }$, respectively. $\sigma_{I_{j}}$ and $\sigma_{I_{j}^{\prime }}$ represent the variances of $I_{j}$ and $I_{j}^{\prime }$, respectively, while $\sigma_{I_{j},I_{j}^{\prime }}$ is the covariance between $I_{j}$ and $I_{j}^{\prime }$. $c_{1}$ and $c_{2}$ are constants.

The Mean Squared Error (MSE) is employed to quantify the brightness difference between the low-light image and the enhanced image. When MSE is used as a loss function, it is defined by Equation (15).

(15) $$L_{MSE}=\sum_{j=1}^{J}\sum_{h=1}^{H}\sum_{w=1}^{W}\frac{1}{W\cdot H}{\left[I_{j}\left(w,h\right)-I_{j}^{\prime }\left(w,h\right)\right]}^{2}]$$

where *H* and *W* represent the height and width of the image, respectively. $I_{j}\left(w,h\right)$ represents the brightness of the pixel at coordinates (w,h) in the image $I_{j}$.

The total loss function includes $L_{SSIM}$ and $L_{MSE}$, as shown in the following Equation (16).

(16) $$L=\lambda_{MSE}\cdot L_{MSE}+\lambda_{SSIM}\cdot L_{SSIM}$$

where $\lambda_{MSE}$ and $\lambda_{SSIM}$ represent the weights of the loss functions $L_{MSE}$ and $L_{SSIM}$, which are set to 20 and 5, respectively, in this paper.

## 4. Experiments

### 4.1. Experimental Details

#### 4.1.1. Implementation Details

All experiments in this paper were conducted on a platform with the Ubuntu 22.04 operating system, and the hardware environment consists of Intel i7-13700K CPU, NVIDIA RTX 4090 24 GB GPU, 32 GB RAM. The Priori DCE is implemented based on the PyTorch framework. To accelerate the convergence of the model training process, the optimizer is set to AdamW [44] with a weight decay of 1e−4, and the batchsize, numworkers, epochs, and initial learning rate are set to 1, 4, 120, and 2e−5, respectively. The learning rate decay strategy is that reducing the learning rate to 0.8 of its previous value every 10 epochs.

#### 4.1.2. Datasets

In recent years, several low-light image datasets have been proposed, which are mainly classified into two categories: paired datasets (with reference) and unpaired datasets (without reference).

The paired datasets used in the experiments include LOLv1 [13], LOLv2 [14], and LSRW [15]. LOLv1 is the first dataset that includes images from real-world scenarios and contains 485 training pairs and 15 validation pairs. The LOLv2 dataset comprises two subsets: LOLv2-Real and LOLv2-Synthetic. LOLv2-Real contains 689 training pairs and 100 validation pairs, while LOLv2-Synthetic includes 900 training pairs and 100 validation pairs. The LSRW dataset also includes two subsets: LSRW-Huawei and LSRW-Nikon. LSRW-Huawei, collected with a Huawei phone, consists of 2450 training pairs and 30 validation pairs. LSRW-Nikon, collected with a Nikon camera, contains 3150 training pairs and 20 validation pairs. The unpaired datasets used in the experiments are DICM [45], LIME [8], MEF [46], and NPE [41], which contain 69, 10, 17, and 85 low-light images, respectively.

#### 4.1.3. Metrics

The evaluation on paired datasets primarily relies on two metrics: PSNR (Peak Signal-to-Noise Ratio; higher is better) and SSIM (Structural Similarity; higher is better). PSNR quantifies the ratio of signal to noise in an image, while SSIM measures the similarity between the enhanced image and the reference image in terms of luminance, contrast, and structure. Notably, the unit of SSIM is expressed as a percentage (%) in the experiments. For unpaired datasets, the evaluation primarily utilizes NIQE [47] (Natural Image Quality Evaluator; lower is better). On the one hand, the metrics for each image in the dataset are computed and averaged to assess the model’s performance, denoted as ${\mathrm{PSNR}}_{m}$, ${\mathrm{SSIM}}_{m}$, and ${\mathrm{NIQE}}_{m}$, respectively. On the other hand, to evaluate the stability of enhancement performance, we use the standard deviation instead of the mean, represented as ${\mathrm{PSNR}}_{s}$, ${\mathrm{SSIM}}_{s}$, and ${\mathrm{NIQE}}_{s}$, respectively.

### 4.2. Ablation Study

#### 4.2.1. The Impact of Loss Function Strategies

In order to enhance the model’s capability for low-light image enhancement, the loss function used during training is composed of two components: $L_{MSE}$ and $L_{SSIM}$. To evaluate the impact of $L_{MSE}$ and $L_{SSIM}$, the model is first trained using $L_{MSE}$ as the sole loss function. Subsequently, both $L_{MSE}$ and $L_{SSIM}$ are employed together as the loss function for training.

Table 1 shows the quantization performance of Priori DCE trained with different loss function strategies on the datasets LOLv1, LOLv2-Real, and LOLv2-Synthetic. As can be seen from Table 1, compared to using $L_{MSE}$ alone as the loss function, the inclusion of $L_{SSIM}$ improves the Priori DCE’s enhancement capability to some extent.

**Figure 8:**
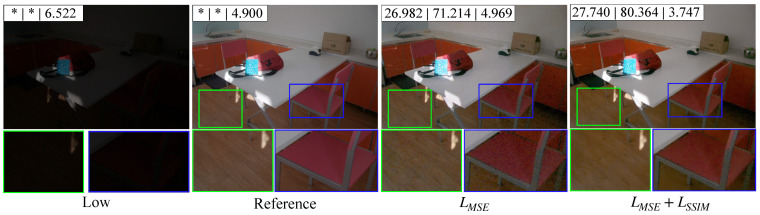
The visualization of the enhancement results of Priori DCE on the same low-light image under different loss function strategies. * indicates that the value does not exist.

Figure 8 visually presents the enhancement results using different loss function strategies. As shown, when $L_{MSE}$ is used alone, both the brightness and visual quality of the enhanced image improve significantly. However, some blurring is observed on the surfaces of the wooden floor and the chair. Incorporating $L_{SSIM}$ into the loss function alleviates this shortcoming, leading to more natural texture transitions. Notably, when both $L_{MSE}$ and $L_{SSIM}$ are combined, the enhanced image outperforms the reference image in terms of the NIQE metric. In the reference image, the texture transition on the wooden floor is relatively sharp, whereas in the enhanced image, the texture becomes noticeably softer, improving the overall visual quality.

#### 4.2.2. The Impact of Hyperparameter *k*

**Figure 9:**
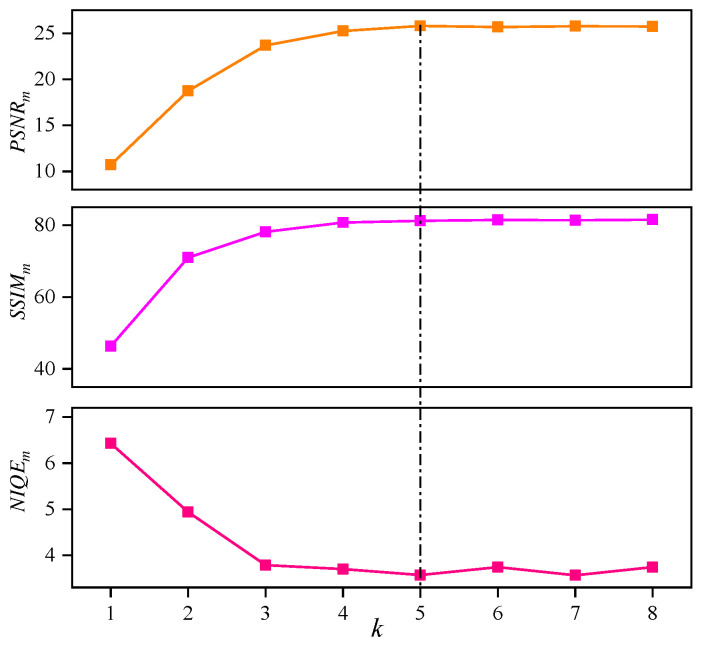
As *k* gradually increases from 1 to 8, the enhancement performance of the corresponding Priori DCE on the LOLv1 dataset.

**Figure 10:**
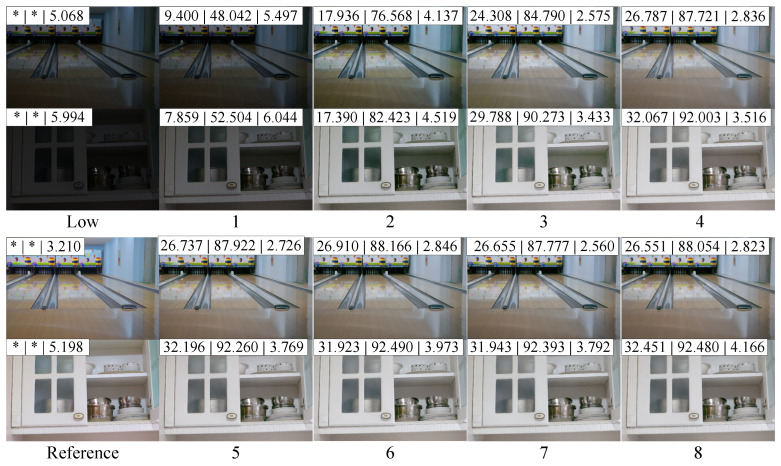
As *k* gradually increases from 1 to 8, the enhanced images generated by corresponding Priori DCE.

As the hyperparameter *k* (the number of iterations of the basis function) increases, the range of pixel’s brightness *x* that is mapped becomes broader, allowing the enhanced pixel to either become brighter or darker. Figure 9 demonstrates the enhancement performance of the Priori DCE with different *k* on the LOLv1 dataset. The *x*-axis represents the hyperparameter *k*, and the *y*-axis shows the corresponding evaluation metrics (${\mathrm{PSNR}}_{m}$, ${\mathrm{SSIM}}_{m}$, and ${\mathrm{NIQE}}_{m}$). It is evident that as *k* increases from 1 to 5, the Priori DCE’s enhancement performance gradually improves, after which it stabilizes. Figure 10 visually presents the enhancement results of the Priori DCE on two low-light images (Bowling Alley and Kitchen Cabinet) with different *k*. The numbers (a|b|c) at the top of the images indicate the corresponding PSNR, SSIM, and NIQE, respectively. It can be observed that when *k* is 1, although the brightness of the enhanced image improves relative to the original low-light image, the issue of underexposure remains. This is due to the limited range of enhanced pixels mapped by the corresponding enhancement function. As *k* increases from 1 to 5, this issue is alleviated. The corresponding enhanced images gradually brighten, and the image quality significantly improves, with the NIQE decreasing from 5.497 and 6.044 to 2.726 and 3.769, respectively. However, the increase in *k* does not result in indefinite improvement in enhancement performance. Once *k* exceeds 5, the brightness and quality of the enhanced images stabilize, with the NIQE changing from 2.726 and 3.769 to 2.823 and 4.166, respectively.

#### 4.2.3. Model Structure

To verify the role of different modules in the Priori DCE proposed in this paper, we use a plain model without any special modules as the baseline and progressively incorporate various modules. Training and testing are performed on the LOLv1, LOLv2-Real, and LOLv2-Synthetic datasets, with quantitative results presented in Table 2. As shown in Table 2, compared to the baseline, the inclusion of the priori channels improves model performance across all three datasets. The priori channels specifies the direction of enhancement for the low-light image, so the enhanced image is more similar to the reference image. Subsequently, incorporating GA further boosts performance, as the enhancement of each pixel in the low-light image is no longer confined to local regions but can be inferred globally. Finally, the addition of the priori probability further improves enhancement performance by providing adaptive priori enhancement probability that varies based on the brightness of pixels.

#### 4.2.4. The Impact of Scale Factor γ

**Figure 11:**
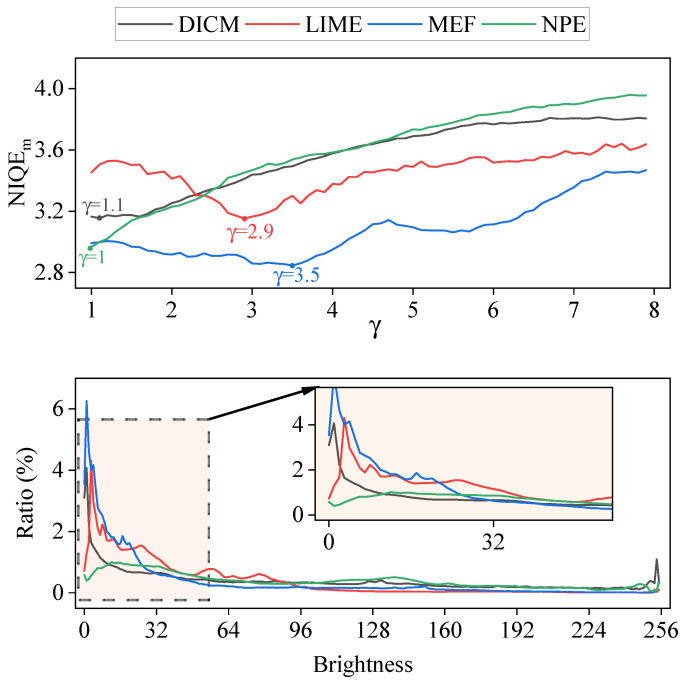
(**Top**): the enhancement performance of Priori DCE with different scale factor γ on datasets DICM, LIME, MEF and NPE, respectively. (**Bottom**): the ratio of pixels with different brightness in these four datasets.

To investigate the effect of the scale factor γ on enhancement performance, experiments were conducted on the datasets DICM, LIME, MEF, and NPE, with the results presented in Figure 11 (top). As shown, optimal performance is achieved on the datasets DICM, LIME, MEF, and NPE when γ is set to 1.1, 2.9, 3.5, and 1, respectively. This is due to the variability in the brightness of different low-light images, as well as the non-uniqueness of the reference images corresponding to them. Therefore, the corresponding priori channels are also different. Figure 11 (bottom) shows the proportion of pixels with different brightness in these four datasets. It can be seen that the brightness in the LIME and MEF datasets are mainly concentrated in the low-light regions. Therefore, to achieve a good enhancement effect, a larger γ is required. In contrast, the brightness distribution in the DICM and NPE datasets is more balanced, so a smaller γ value is sufficient.

**Figure 12:**
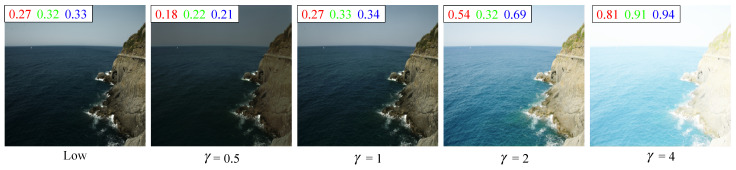
The enhanced result of priori DCE when the γ was 0.5, 1, 2, and 4, respectively. The numbers in red, green, and blue represent the average on the corresponding channel.

A γ value of 0.5, 1, 2, and 4 indicates that the brightness of the enhanced image is 0.5 times, 1 times, 2 times, and 4 times that of the original low-light image, and Figure 12 visualizes the enhancement results corresponding to these γ, with different colored numbers representing the average brightness on the corresponding channels. As shown in Figure 12, the average brightness of the three channels of the original low-light image is 0.27, 0.32, and 0.33, respectively. The three channels of the enhanced image corresponding to a γ of 0.5 are 0.18, 0.22, and 0.21, respectively, which is significantly lower than that of the original low-light image. The enhanced image when the γ is 1 is almost the same brightness as the original low-light image. When the γ is 2, the brightness of the enhanced image is close to 0.5, and the quality of the enhanced image is also the best. When the γ is 4, the corresponding enhanced image is significantly overexposed. As can be seen from the above, the output of the model can be adjusted by adjusting the value of γ, so that for different low-light images, enhanced images with different brightness can be generated.

### 4.3. Performance Evaluation

#### 4.3.1. Reference Evaluation

The LOLv1 [13], LOLv2 [14], and LSRW [15] datasets are currently popular paired datasets. In this section, we evaluate several recent SOTA methods along with Priori DCE on these paired datasets, with the results presented in Table 3. As shown, EnlightenGAN, MELLEN-IC, Retinexformer, LLFlow, and Priori DCE achieve the best performance, with Priori DCE demonstrating the strongest enhancement performance. Notably, when comparing Priori DCE with the second-best performing Retinexformer on the LOLv2-Synthetic dataset, the ${\mathrm{PSNR}}_{m}$ and ${\mathrm{SSIM}}_{m}$ improve from 25.67 and 92.82 to 29.49 and 93.6, respectively, while the ${\mathrm{NIQE}}_{m}$ for image quality decreases slightly from 3.94 to 3.91. Additionally, Table 3 reports the ${\mathrm{NIQE}}_{m}$ for both the low-light image (Low) and reference image (Reference). It is evident that some methods, such as MELLEN-IC, Retinexformer, LLFlow, and Priori DCE, produce enhanced images with ${\mathrm{NIQE}}_{m}$ lower than those of the reference images. This can be attributed to the fact that low-light image enhancement models are trained on large datasets, most of which contain high-quality reference images (lower NIQE). Consequently, these models not only learn how to enhance brightness but also improve the overall image quality.

Table 3 also shows the complexity and running speed of different models. It can be seen that DeepUPE has a very small number of parameters, and its running speed even reaches 2426, and in exchange, its enhanced quality is terrible. Since the Priori DCE model introduced the attention mechanism GA, its running speed was reduced from 24 to 11.67 compared to Zero DCE, and in exchange, the enhancement quality of Zero DCE was also weaker than that of Priori DCE. It can be seen that although the enhanced quality of Priori DCE has been enhanced, how to improve the operation efficiency is an important research direction in the future.

**Figure 13:**
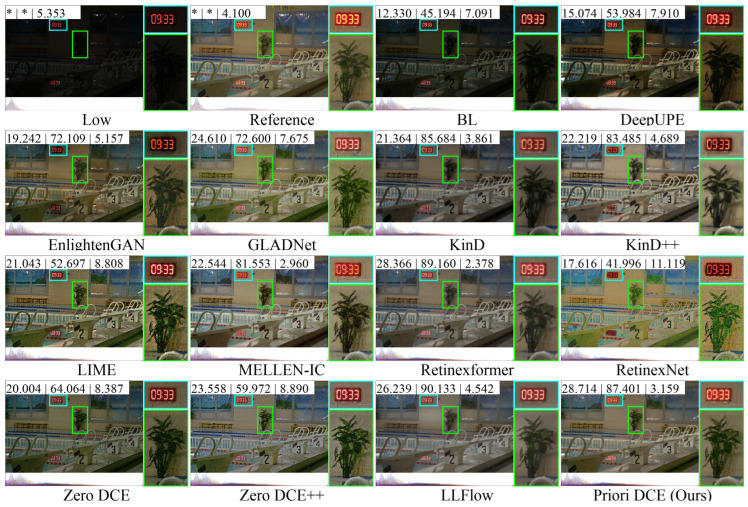
The enhancement visualization on the LOLv1 dataset.

**Figure 14:**
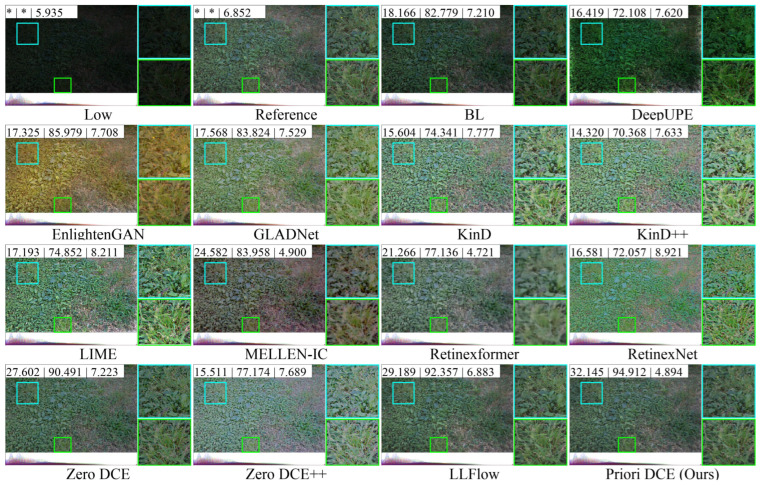
The enhancement visualization on the LOLv2-Real dataset.

**Figure 15:**
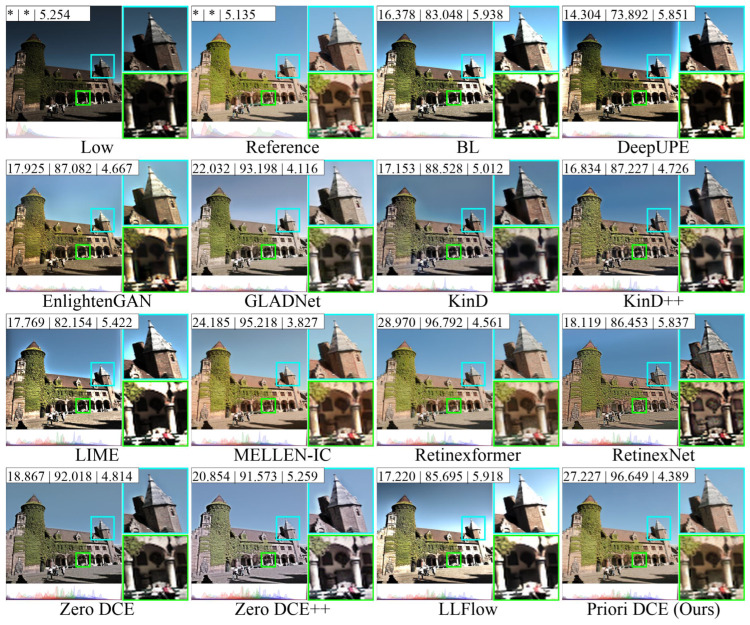
The enhancement visualization on the LOLv2-Synthetic dataset.

**Figure 16:**
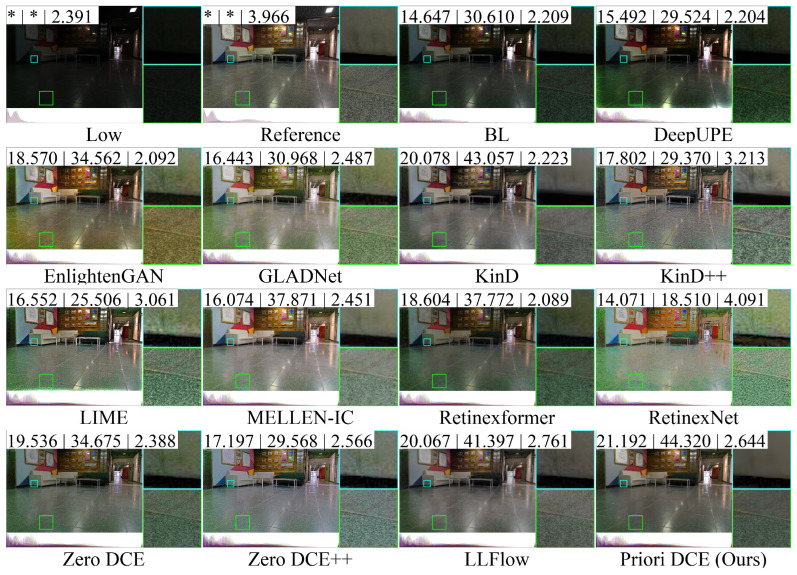
The enhancement visualization on the LSRW-Huawei dataset.

**Figure 17:**
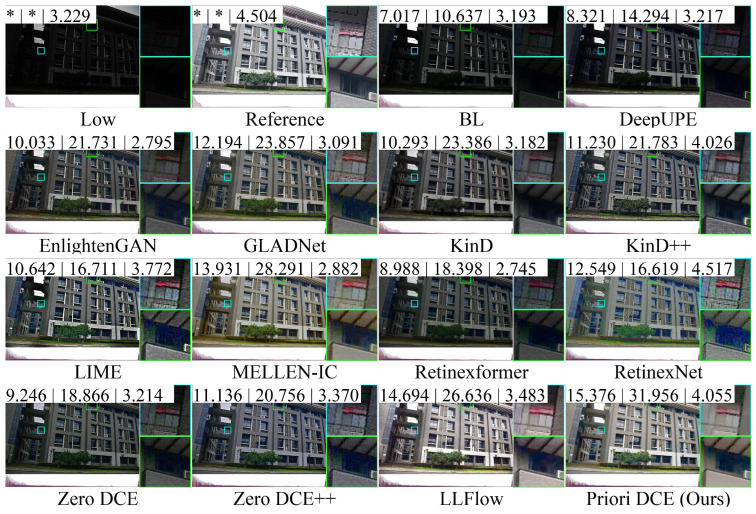
The enhancement visualization on the LSRW-Nikon dataset.

Figure 13, Figure 14, Figure 15, Figure 16 and Figure 17 visually display the enhancement results on different datasets. The numbers (a|b|c) at the top of these images correspond to the PSNR, SSIM, and NIQE values of the respective images. The histograms at the bottom represent the frequency distribution of brightness in the R, G, and B channels for the corresponding images.

Figure 13 shows the low-light image of a swimming pool scene from the LOLv1 dataset and these corresponding enhanced images generated by several SOTA methods. As shown in the figure, the brightness of BL, DeepUPE, and Zero DCE is relatively low. Correspondingly, the brightness distribution histograms are concentrated primarily in the lower brightness range on the left. The enhancement quality of these three methods is even worse than that of the original low-light image. In contrast, EnlightenGAN, GLADNet, LIME, RetinexNet, and Zero DCE++ exhibit significant improvements in brightness. However, while the brightness was increased, different levels of noise were also introduced, with the noise in the trees and the surrounding areas of the enhanced image being particularly noticeable. RetinxeNet even encountered serious stylization issues. This led to a decrease in the enhancement quality. The performance of KinD, KinD++, MELLEN-IC, Retinexformer, LLFlow, and Priori DCE is relatively better. In the reference image, the text on the clock is brighter. In the enhanced images from KinD, KinD++, MELLEN-IC, Retinexformer, and LLFlow, there is a noticeable color shift in the clock text. Meanwhile, thanks to the inclusion of priori channels, The enhancement effect of Priori DCE on these texts in the clock is closest to that of reference.

Figure 14 shows the low-light image of a grassland scene from the LOLv2-Real dataset and the corresponding enhanced images generated by several SOTA methods. BL and DeepUPE still have weak brightness, and their corresponding frequency distribution histograms are mainly concentrated in the low-brightness region. EnlightenGAN is notably yellowish. In fact, the brightness of the reference image is also low, but the brightness of these enhanced images generated by GLADNet, KinD, KinD++, RetinexNet, and Zero DCE++, is significantly increased, even surpassing the brightness of the reference. The enhancement quality of these methods is therefore not satisfactory, as the NIQE is greater than the reference and even exceeds 7. In comparison, Retinexformer, LLFlow, and Priori DCE are closer to the reference. However, based on the enhancement results of the grass area within the cyan and green boxes in the image, Retinexformer images are relatively blurry, while LLFlow and Priori DCE images are more satisfactory.

Figure 15 shows the low-light image of building from the dataset LOLv2-Synthetic and these corresponding enhanced images generated by several SOTA methods. It can be observed that the brightness of the reference is high, and the frequency distribution histogram is more evenly distributed. From the rooftop area within the cyan box, the colors in the reference image are predominantly composed of gray and warm yellow tones. Among these methods, only MELLEN-IC, Retinexformer, and Priori DCE are closest to the reference, while the colors of other methods show significant deviations. In BL, LIME, and LLFlow, the transition between the roof and the sky even shows a noticeable white area.

Figure 16 shows the low-light image of the rest room scene in the LSRW-Huawei dataset and these corresponding enhanced images generated by several SOTA methods. The brightness of BL and DeepUPE is relatively weak. EnlightenGAN, GALDNet, KinD++, and LIME exhibit significant color deviation and generate excessive noise. RetinexNet still suffers from stylization issues. MELLEN-IC and Zero DCE++ exhibit higher brightness compared to the reference, which results in lower PSNR and SSIM values for these methods. This occurs because these methods focus solely on enhancing the low-light image without accounting for the reference. Among these methods, Priori DCE most closely matches the reference image, successfully improving both image quality and brightness.

Figure 17 shows the low-light image of multi-floor building from the LSRW-Nikon dataset and the corresponding enhanced images generated by several SOTA methods. It is evident that the building areas in the reference image are relatively bright. Among these methods, only LLFlow and Priori DCE yield satisfactory results in both brightness and enhancement quality. Other methods either produce insufficient brightness or exhibit noticeable color distortions. In the window highlighted by the cyan box, Priori DCE introduces more noise, resulting in lower enhancement quality compared to LLFlow.

The enhancement results above indicate that Retinexformer, LLFlow, and Priori DCE achieve good enhancement quality. However, the performance of Retinexformer and LLFlow is unstable. For instance, Retinexformer produces darker enhanced image on the LSRW-Nikon dataset, while LLFlow introduces white transitions in the image from the LOLv2-Synthetic dataset. In contrast, Priori DCE demonstrates a clear advantage in both enhancement quality and stability across these datasets.

#### 4.3.2. Unreference Evaluation

DICM, LIME, MEF, and NPE are currently popular datasets without reference. Similar to Section 4.3.1, we conduct experiments on these datasets using the same SOTA methods, with the results presented in Table 4. Since these datasets do not have reference images, we use ${\mathrm{NIQE}}_{m}$ and ${\mathrm{NIQE}}_{s}$ as evaluation metrics. Additionally, we calculate the average results across different datasets, as shown in the avg column of Table 4. As shown in Table 4, the top three models are EnlightenGAN, MELLEN-IC, and Priori DCE. Among these, Priori DCE shows a clear advantage in both enhancement quality and stability. Compared to MELLEN-IC, which ranks second in the avg metric, Priori DCE reduces the ${\mathrm{NIQE}}_{m}$ and ${\mathrm{NIQE}}_{s}$ scores from 3.15 and 0.914 to 3.031 and 0.839, respectively. This demonstrates the effectiveness and stability advantages of Priori DCE.

**Figure 18:**
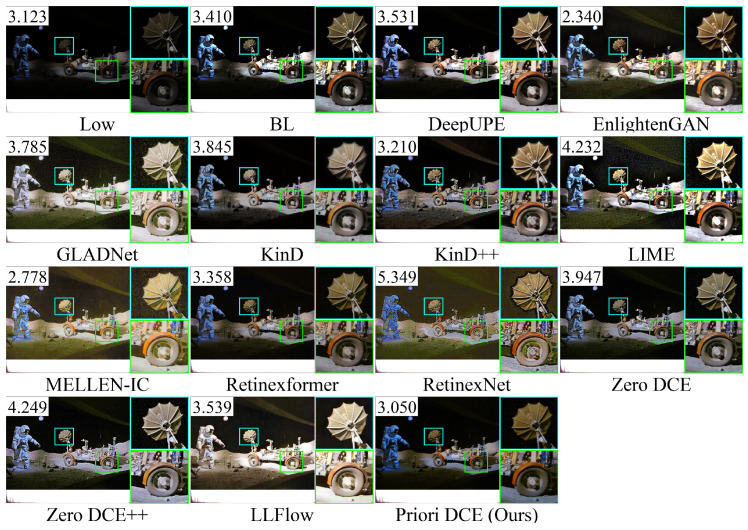
The enhancement visualization on the DICM dataset.

Figure 18 shows the low-light image of the lunar landing scene from the DICM dataset and the corresponding enhanced images generated by several SOTA methods. It can be observed that RetinexNet still exhibits obvious stylization, while the astronaut’s color in LLFlow and GLADNet has turned white. The top three methods with the best enhancement quality are Priori DCE, MELLEN-IC, and EnlightenGAN, with corresponding NIQE values of 3.05, 2.778, and 2.34, respectively. From the zoomed-in views of the radar and wheels, it can be seen that EnlightenGAN provides the highest enhancement quality, preserving rich details while increasing brightness. Similar to EnlightenGAN, MELLEN-IC increases brightness further but sacrifices some details. Priori DCE, while retaining details, has insufficient brightness.

**Figure 19:**
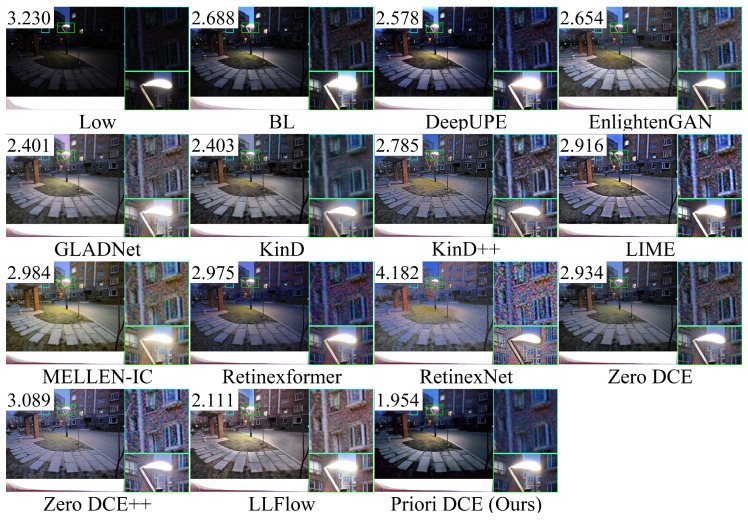
The enhancement visualization on the LIME dataset.

Figure 19 shows the low-light image of a streetlight scene from the LIME dataset and the corresponding enhanced images generated by several SOTA methods. From the zoomed-in views of the buildings and streetlight, it can be seen that GLADNet, KinD++, LIME, RetinexNet, and Zero DCE exhibit the most significant noise. LLFlow produces the brightest image with only a small amount of noise. Priori DCE introduces the least noise around the streetlight and provides the most natural transition between the streetlight and its surrounding environment.

**Figure 20:**
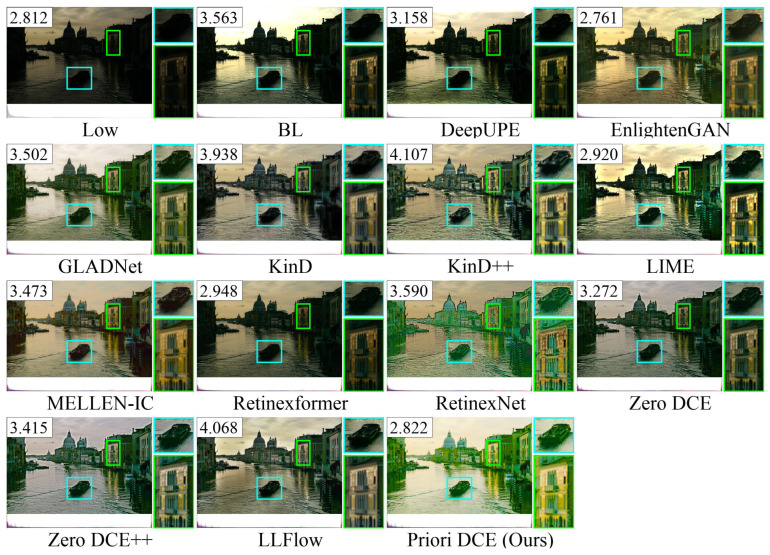
The enhancement visualization on the MEF dataset.

Figure 20 shows the low-light image of the venice from the MEF dataset and these corresponding enhanced images generated by several SOTA methods. Notably, Priori DCE achieves the highest brightness while maintaining strong enhancement quality. Furthermore, the enhancement quality of EnlightenGAN, LIME, and Retinexformer is also relatively high, primarily due to their lower brightness in these enhanced images.

**Figure 21:**
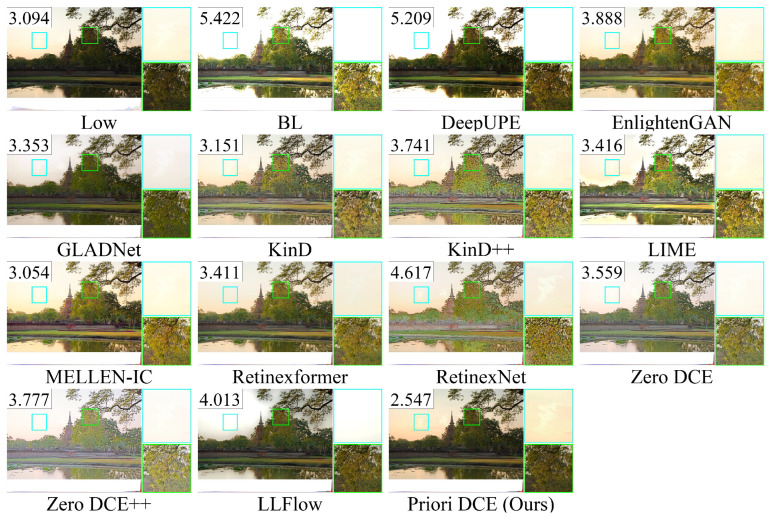
The enhancement visualization on the NPE dataset.

Figure 21 shows the low-light image of the forest from the NPE dataset and the corresponding enhanced images generated by several SOTA methods. The zoomed-in view of the sky reveals that BL, DeepUPE, GLADNet, Zero DCE++, and LLFlow largely overlook its texture, whereas RetinexNet and Priori DCE provide the most effective enhancement of the sky’s texture. In the zoomed-in view of the forest, the brightness of DeepUPE, GLADNet, Retinexformer, LLFlow, and Priori DCE are relatively low, while other methods are noticeably overexposed. KinD appears somewhat blurred, neglecting the texture. RetinexNet appears highly unnatural.

## 5. Conclusions

This paper incorporates priori knowledge into low-light image enhancement method through the use of priori channels and priori enhancement probability and names the method Priori DCE. The priori channels enable the adjustable brightness of the enhanced image, while the priori enhancement probabilities are adaptively adjusted according to the brightness of individual pixels. Additionally, the GA module is integrated to facilitate interactions between pixels in the enhanced image, promoting visual balance in the enhanced image. Experimental results demonstrate the superior performance of the proposed Priori DCE. However, due to the introduction of GA modules, on the one hand, it improves the enhanced quality of the model, and on the other hand, it also reduces the operational efficiency. How to solve this problem is one of the main research directions in the future.

## Figures and Tables

**Table 1:** The enhancement performance of Priori DCE under different loss function strategies.

Loss	LOLv1			LOLv2-Real			LOLv2-Synthetic		
	${\mathrm{PSNR}}_{m}$	${\mathrm{SSIM}}_{m}$	${\mathrm{NIQE}}_{m}$	${\mathrm{PSNR}}_{m}$	${\mathrm{SSIM}}_{m}$	${\mathrm{NIQE}}_{m}$	${\mathrm{PSNR}}_{m}$	${\mathrm{SSIM}}_{m}$	${\mathrm{NIQE}}_{m}$
$L_{MSE}$	25.015	75.102	4.444	25.825	73.812	5.357	28.326	91.380	3.869
$L_{MSE}$ + $L_{SSIM}$	25.775	81.222	3.572	26.828	80.791	4.381	29.488	93.598	3.911

**Table 2:** The enhancement performance of the models after adding different modules to the baseline. Red font indicates that the item is optimal for the column.

Priori Channels	GA	Priori Probability	LOLv1			LOLv2-Real			LOLv2-Synthetic		
			${\mathrm{PSNR}}_{m}$	${\mathrm{SSIM}}_{m}$	${\mathrm{NIQE}}_{m}$	${\mathrm{PSNR}}_{m}$	${\mathrm{SSIM}}_{m}$	${\mathrm{NIQE}}_{m}$	${\mathrm{PSNR}}_{m}$	${\mathrm{SSIM}}_{m}$	${\mathrm{NIQE}}_{m}$
baseline			21.002	77.737	3.675	22.028	76.907	4.290	21.495	89.793	3.879
✓			22.408	76.945	3.823	23.877	76.815	4.382	22.453	90.580	3.818
✓	✓		24.376	79.162	3.819	25.074	78.529	4.394	29.115	93.661	3.895
✓	✓	✓	25.775	81.222	3.572	26.828	80.791	4.381	29.488	93.598	3.911

**Table 3:** The enhancement performance of different SOTA models on the LOLv1, LOLv2, and LSRW datasets. The best and second-best performances are represented in red and blue, respectively.

Method	Complexity			LOLv1			LOLv2_Real			LOLv2_Synthetic			LSRW_Huawei			LSRW_Nikon		
	MACs (G)	Params (M)	FPS	${\mathrm{PSNR}}_{m}$	${\mathrm{SSIM}}_{m}$	${\mathrm{NIQE}}_{m}$	${\mathrm{PSNR}}_{m}$	${\mathrm{SSIM}}_{m}$	${\mathrm{NIQE}}_{m}$	${\mathrm{PSNR}}_{m}$	${\mathrm{SSIM}}_{m}$	${\mathrm{NIQE}}_{m}$	${\mathrm{PSNR}}_{m}$	${\mathrm{SSIM}}_{m}$	${\mathrm{NIQE}}_{m}$	${\mathrm{PSNR}}_{m}$	${\mathrm{SSIM}}_{m}$	${\mathrm{NIQE}}_{m}$
Low						5.72			6.01			4.09			3.16			3.45
Reference						4.25			4.73			4.19			3.44			4.24
BL [48]	150.799	1.606	146.895	10.31	40.13	7.31	12.89	43.53	7.73	13.58	61.44	4.74	11.78	31.24	3.06	13.43	36.19	3.85
DeepUPE [18]	45.935	0.079	2426.394	12.71	45.04	7.79	14.60	47.02	8.23	13.82	60.50	4.37	13.63	36.25	3.00	13.36	35.97	3.64
EnlightenGAN [24]			42.983	17.48	65.15	4.89	18.64	67.67	5.50	16.57	77.15	3.83	17.85	48.92	2.94	15.92	42.09	3.18
GLADNet [35]	200.6	12.15	79.839	19.72	68.20	6.80	19.82	68.47	7.73	18.11	82.59	3.99	19.00	49.45	2.96	16.63	44.07	3.36
KinD [19]	61.01	114.35	24.385	17.64	77.13	3.90	20.58	81.78	4.14	17.27	75.78	4.25	17.03	49.88	2.64	15.47	44.04	3.46
KinD++ [7]	1050	17.42	14.844	17.75	75.82	4.01	17.66	76.09	4.20	17.48	78.57	4.76	16.97	41.15	3.02	14.74	36.80	3.72
LIME [8]			3.463	16.05	48.60	8.79	17.16	48.02	9.31	16.37	73.74	4.76	17.13	39.31	3.44	14.64	34.99	3.61
MELLEN-IC [25]	2532	8.275	1.432	17.23	75.44	3.31	20.75	78.98	3.32	21.57	88.08	3.98	18.22	53.48	2.64	16.71	45.08	3.41
Retinexformer [42]			9.293	25.15	84.34	2.97	22.79	83.86	3.59	25.67	92.82	3.94	16.25	49.48	2.60	15.56	42.38	3.27
RetinexNet [13]			9.342	16.77	42.50	9.73	16.10	40.71	10.56	17.14	75.64	5.69	16.82	38.50	4.33	13.49	28.94	4.27
Zero DCE [1]	517.129	28.539	24.091	14.86	56.24	8.22	18.06	57.95	8.77	17.76	81.40	4.36	16.41	46.19	3.15	15.05	41.37	3.40
Zero DCE++ [26]	0.109	0.594	123.976	17.04	56.25	8.46	18.14	55.18	9.06	18.64	83.52	4.55	18.12	45.55	3.27	15.10	39.20	3.56
LLFlow [49]			0.761	24.06	86.02	4.07	26.43	90.26	4.53	19.22	82.41	4.66	20.09	55.07	2.88	16.88	45.66	3.73
Priori DCE	834.2	36.21	11.673	25.77	81.22	3.57	26.83	80.79	4.38	29.49	93.60	3.91	21.39	56.76	3.13	18.33	48.52	3.40

**Table 4:** The enhancement performance of different SOTA models on the DICM, LIME, MEF, and NPE datasets. The best and second-best performances are represented in red and blue, respectively.

Method	${\mathrm{NIQE}}_{m}$					${\mathrm{NIQE}}_{s}$				
	DICM	LIME	MEF	NPE	avg	DICM	LIME	MEF	NPE	avg
Low	3.317	3.566	3.256	3.187	3.332					
BL [48]	4.078	4.216	3.383	4.424	4.025	1.534	1.933	0.665	1.877	1.502
DeepUPE [18]	3.542	3.793	3.199	3.591	3.531	0.985	2.094	0.546	1.258	1.221
EnlightenGAN [24]	3.056	3.380	2.895	3.368	3.175	0.823	1.514	0.466	1.241	1.011
GLADNet [35]	3.276	3.902	3.179	3.271	3.407	0.955	2.608	0.625	0.986	1.293
KinD [19]	3.351	4.357	3.378	3.269	3.589	1.017	3.794	0.464	0.934	1.552
KinD++ [7]	3.280	4.853	3.471	3.636	3.810	1.000	4.466	0.473	1.220	1.790
LIME [8]	3.471	3.835	3.488	3.470	3.566	1.179	2.364	0.804	1.258	1.401
MELLEN-IC [25]	2.911	3.503	3.097	3.087	3.150	0.784	1.673	0.415	0.783	0.914
Retinexformer [42]	3.353	3.705	3.139	3.174	3.343	0.972	1.468	0.753	1.014	1.052
RetinexNet [13]	4.315	4.916	4.904	4.388	4.631	1.715	3.557	1.475	1.496	2.061
Zero DCE [1]	3.430	3.786	3.309	3.433	3.489	1.195	2.093	0.861	1.223	1.343
Zero DCE++ [26]	3.543	4.092	3.568	3.603	3.701	1.259	2.489	0.953	1.249	1.488
LLFlow [49]	3.368	3.891	3.515	3.556	3.583	0.885	2.007	0.542	0.877	1.078
Priori DCE (Ours)	3.155	3.153	2.848	2.968	3.031	0.872	1.174	0.603	0.708	0.839

## Data Availability

The datasets used in this work are openly available.
